# Cushingoid clinical features in a pediatric patient taking a natural supplement: Buyer beware

**DOI:** 10.1016/j.jdcr.2023.08.035

**Published:** 2023-09-07

**Authors:** Advika Dani, Kaleo Ede, Harper Price, Vanessa Gildenstern

**Affiliations:** a Division of Dermatology, Phoenix Children’s Hospital, Phoenix, Arizona; b The Johns Hopkins University School of Medicine, Baltimore, Maryland; c Department of Child Health, University of Arizona College of Medicine – Phoenix, Phoenix, Arizona; d Division of Rheumatology, Phoenix Children’s Hospital, Phoenix, Arizona

**Keywords:** Cushing syndrome, glucocorticoids, herbal supplements, natural supplements, pediatrics

## Introduction

This natural supplement (Artri-King, manufactured by NaturaMex) is a Mexican-manufactured dietary supplement advertised for joint pain and arthritis. Ingredients listed include collagen, vitamin C, Omega 3s, glucosamine, chondroitin sulfate, and turmeric. An April 2022, US Food and Drug Administration Public Notice alerted consumers that this product contains unregulated and unlabeled amounts of diclofenac and dexamethasone.^1^ The Food and Drug Administration’s notification spurred a nationwide recall of this product. Patients taking this medication are at risk of iatrogenic Cushing syndrome (CS) due to inadvertent chronic corticosteroid use. Exogenous corticosteroids cause increased rates of gluconeogenesis, glycogenolysis, and increased insulin resistance, which can lead to severe health consequences. The most common cause of CS is iatrogenic in adults; however, corticotropin-secreting pituitary tumors have been reported as the most common cause of CS in children older than age 6.^2^^,^^3^ Case reports of iatrogenic CS due to intranasal, topical, oral and ocular dexamethasone have been described in infants and children.^4^ Along with height deceleration and weight gain, children with CS can also present with headaches, bruising, fungal infections, bone fractures, irritability, and fatigue.^3^

While there are few reported cases of CS from this supplement in adults, no pediatric cases have been reported thus far.^5^ We report a case of a pediatric patient with Cushingoid clinical features with chronic use of this supplement.

## Case report

**Fig 1:**
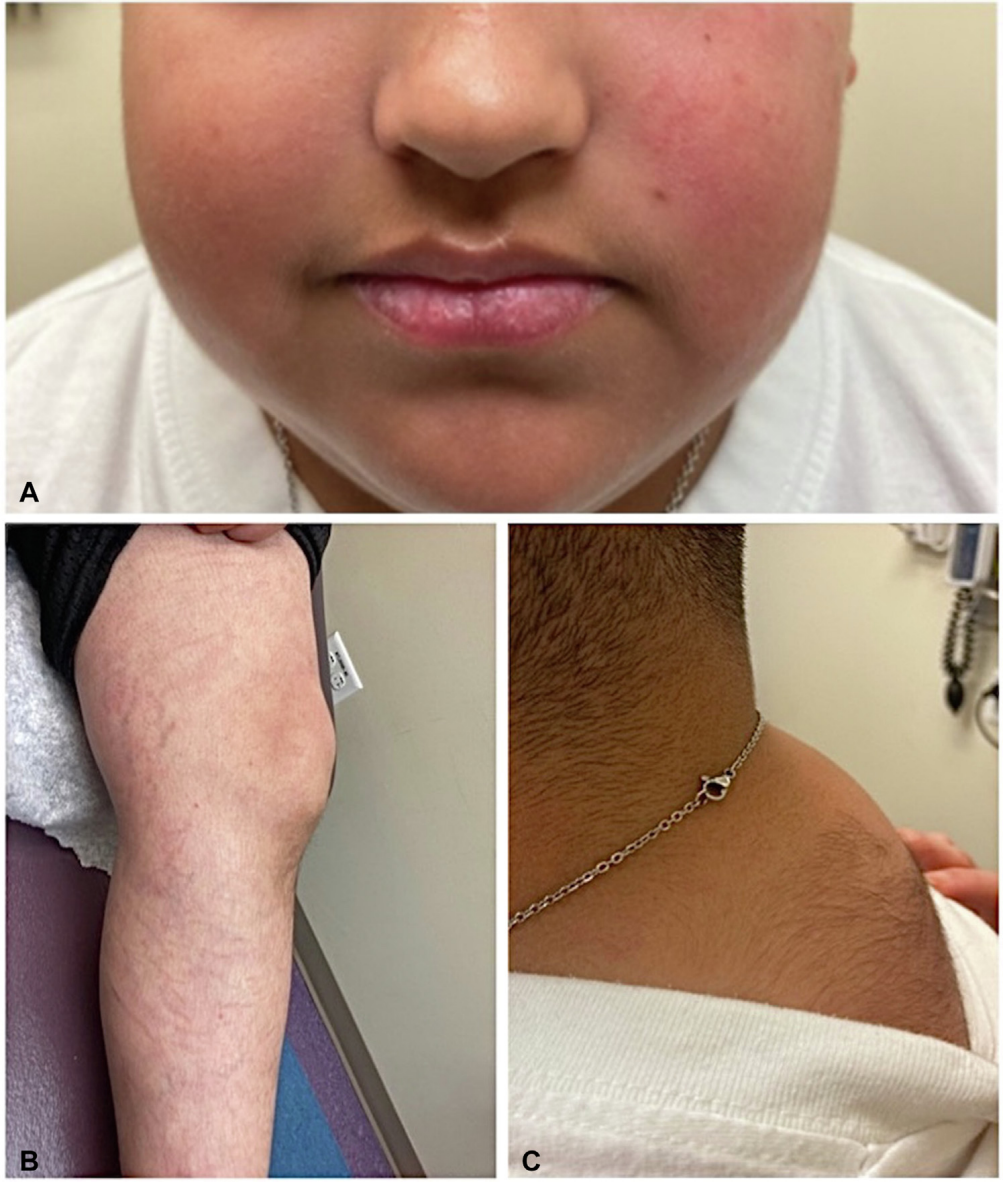
Cushingoid features observed in a 12-year-old male taking natural supplement. **A,** Round facies and facial plethora. **B,** Striae observed on the *left* proximal and distal *lower* extremity. **C,** Prominent *upper* back hump and hypertrichosis of the back.

A 12-year-old boy with a remote history of cutaneous small vessel vasculitis and undifferentiated juvenile idiopathic arthritis presented for evaluation of facial dermatitis in the pediatric dermatology clinic. Moon facies, facial plethora, a prominent soft tissue upper back hump with hypertrichosis, and pink striae along his bilateral proximal and distal legs (Fig 1) were incidentally noted. His medications included aspirin, as well as 2 tablets of the natural supplement daily for the last 10 months. His complete blood count, comprehensive metabolic panel, thyroid stimulating hormone, and other labs were within normal limits, apart from an elevated free T4 of 1.8 (reference value 0.9-1.5 ng/dL). Upon further medication review, prednisone had been discontinued 11 months preceding that evaluation, and no other known steroids were listed in his medicaton profile. This prompted further inquiry into the supplement and the subsequent discovery of the April 2022, Food and Drug Administration Public Notice. The patient’s rheumatologist was informed, and he was referred to endocrinology for concern of iatrogenic CS. At that evaluation, the Cushingoid features persisted. He was found to have a body mass index of 26.8 kg/m^2^, up from 22.9 kg/m^2^ a year earlier, as well as height deceleration, dropping from the 42nd to the eighth percentile over the preceding year.

## Discussion

This case serves as an important reminder to clinicians that over-the-counter supplements should not be disregarded when features of corticosteroid excess are noted, even when culprit ingredients are not listed on the product label. Although typical physiologic striae are commonly seen in adolescents with pubertal onset or significant weight gain, our patient’s additional Cushingoid clinical features and extensive striae heightened our concern. Input from endocrinology is paramount for proper diagnosis and management of iatrogenic CS, since abruptly stopping the causative agent can trigger an adrenal crisis. Pediatric patients with CS need long-term follow-up to assess for adrenal insufficiency, immunosuppression, obesity, insulin resistance, suppression of final height, emotional lability, depression, cognitive development, electrolyte imbalances, hyperglycemia, hypertension, and osteoporosis due to effects of long-term glucocorticoid exposure.^3^ Cardiovascular dysfunction, adiposity of the abdomen, insulin resistance, and psychiatric effects can persist after CS resolves.^3^

Over-the-counter and “natural” supplements may appeal to patients and families because they do not require insurance approval and may be less expensive and perceived as less harmful than prescribed medications. Additionally, families may have barriers to care that make these medications much more accessible than prescription medications. Providers should inquire about supplements when taking a medication history and counsel families about the risks of these unregulated medications. This is especially important for children who may experience irreversible effects on their development, especially if introduced at a young age. This case exemplifies the importance of emphasizing the harms that unregulated supplements can have on this vulnerable population. Of note, information about the exact amount of dexamethasone in each tablet of the natural supplement is not readily available. Wide sample testing would fill this knowledge gap and inform the management of patients taking this supplement.

## Conflicts of interest

None disclosed.
